# Editorial: Cell signaling status alteration in development and disease

**DOI:** 10.3389/fcell.2022.1068887

**Published:** 2022-12-01

**Authors:** Jun Wu, Haipeng Liu, Xiaodong Zhao, Huixiao Hong, Johannes Werner

**Affiliations:** ^1^ School of Life Sciences, East China Normal University, Shanghai, China; ^2^ Shanghai Pulmonary Hospital, School of Medicine, Tongji University, Shanghai, China; ^3^ Shanghai Center for Systems Biomedicine, Shanghai Jiao Tong University, Shanghai, China; ^4^ National Center for Toxicological Research, US Food and Drug Administration, Jefferson, AR, United States; ^5^ Center for Data Processing, University of Tübingen, Tübingen, Germany

**Keywords:** signal pathway, CGAS, STING, multi-omic analyses, Wnt signaling

Organisms respond and adapt to the internal and external environmental changes through various cell signaling. The abnormal alteration of cell-signaling pathways could disrupt cell homeostasis and cause diseases (Ehnnsen and Ditzel, 2021; Nong et al., 2021). For instance, the dysfunction of immune cell signaling has been frequently reported to be pathological hallmarks for various human diseases, such as cancer (Bayik and Lathia, 2021), sepsis (Chen and Wei, 2021), and autoimmune diseases (Hou et al., 2022). Hence, the in-depth studies of alteration of cell signaling status can effectively facilitate our understanding of cell development and provide us specific information for the disease diagnosis and therapy.

Although cell signaling can be measured through development of sequencing technologies and the application of effective bioinformatic tools (Gilbert et al., 2019; Knapp et al., 2019; Ghosh et al., 2021), many questions remain to be answered. Identifying the association between various signaling pathways and human behavior and diseases will help address these questions. Integrative analysis of different signal pathways that enable comprehensive mapping of cell development and the disease occurrence and progression (Chen et al., 2008; Rodchenkov et al., 2020; Zhang-James et al., 2019). In this Research Topic on *Cell Signaling Status Alteration in Development and Disease*, we collected studies providing new insights into the roles of cell signaling in development and disease occurrence and progression. A total of 5 articles, including two reviews, two original researches and one method article, were published in this Research Topic. We summarize and discuss the main findings of these studies in this editorial.

Wnt signaling plays an important role in the mammary gland development and adult homeostasis in virtually all animal species. Willy et al. provided a systematic review on Wnt signaling involved in breast cancer and explored the impact of Wnt signaling alteration on the tumorigenesis and disease occurrence (Abreu de Oliveira et al.). Yang et al. comprehensively reviewed the role of cyclic GMP-AMP synthase (cGAS)—a stimulator of the interferon gene (STING) signaling pathway in various diseases, such as acute injury, pneumonopathy and kidney diseases, providing a theoretical basis for immunotherapy targeting the STING signal pathway (Yang et al.). Ma et al. found that miR-654-5p overexpresses in activated human hepatic stellate cells and TGFβ-treated human hepatic LX-2 cells augmented liver fibrosis in mice that were intraperitoneally injected with CCl4 by targeting the RXRα receptor (Ma et al.). Zhang et al. proposed a robust method to acquire finely resolved transcriptional programs with few cells from snap-frozen or RNAlater-treated clinical tissues that was sufficient enough to resolve even isoforms based on immunofluorescence-guided laser capture microdissection (immuno-LCM-RNAseq). With this method, the researchers were able to analyze transcriptional networks and signaling pathways during development, pathogenesis, and aging of specific cell types within native tissues (Zhang et al.). Lou et al. integrated both immune and hypoxia signaling to establish reliable prognostic signatures for lung adenocarcinoma (LUAD) across different omics data, and provided a robust prognosis predictor for the LUAD patients (Lou et al.).

The studies published in this Research Topic presented a diversity of intriguing and meaningful results covering a range of cell signals, which could facilitate our understanding of development and disease. We hope that this Research Topic will inspire researchers to systematically investigate development and disease progression from the perspective of cell signal in a systematic way.
